# Carcinoma of the Neck of the Uterus Complicating Pregnancy and Labor

**Published:** 1890-01

**Authors:** G. Heinricius


### CARCINOMA OF THE NECK OF THE UTERUS COMPLICATING PREGNANCY AND LABOR.

BY G. HEINRICIUS.

TRANSLATED FROM THE NOUVELLES ARCHIVES D' OBSTETRIQUE ET DE GYNECOLOGIE, BY VIRGIL O. HARDON, M. D.

S. A., aged forty, entered the gynecological service at Helsingfors on the 13th of September, 1884. Menstruated at fourteen, duration of menses one week, flow abundant. Married twelve years, seven children. Leucorrhœa since January, 1884, accompanied by constant pains in the belly. Constipation. The patient could not fix the date of her last menstruation. September 29, the fundus of the uterus is a finger's breadth below the umbilicus. Fœtal movements are distinct but very feeble, and are felt at the fundus. Uterine contractions can be recognized. The fœtal heart beat can be heard low down in the right side. The fœtus is very movable. By internal examination *ballottement* cannot be felt. In the anterior *cul-de-sac* the neck of the uterus found to be irregular, hard and resisting, and presenting clearly an infiltration of the same nature as that which can be felt in the anterior lip, and which extends into the cavity of the cervix as far as the finger can reach. The anterior lip is rounded, somewhat irregular, as large as a pullet's egg but not ulcerated. The posterior lip is swollen and friable, but does not appear to be infiltrated. The *culs-de-sac* are intact. There is no engorgement of the inguinal glands.

The patient entered the hospital on the 5th of February, 1885. The fundus of the uterus was then two finger's breadth below the xiphoid cartilage. The back of the fœtus was to the right, the limbs to the left, the head just above the symphysis pubis. The fœtal heart sounds on the right just below the umbilicus. The inguinal glands were small and hard.

By internal examination the walls of the vagina were found to be smooth; the anterior part of the cervix formed a soft tumor as large as a small apple. The posterior lip was of normal consistence and friable. Through the dilated os could be felt the head of the fœtus enveloped in the membranes. The anterior wall of the cervix felt irregular and rugose. The secretion was bloody but had no odor. On the 6th of February, the patient was carried to the maternity to be confined. Pains commenced on the 7th of February, at five o'clock in the morning, at 7:40 the water broke and at eight a living child, a boy, was born. The presentation was right occipito—anterior. No hemorrhage or other complication during or after labor. Puerperium normal.

She entered the gynecological service on the 25th of April, 1885. On the right side of the upper part of the uterus was a hard, movable tumor as large as a walnut attached to the uterus by a ligamentous cord. On the left side, lower down, was a second tumor, hard and irregular, oblong in shape, movable in the longitudinal axis of the body, not connected with the uterus, perceptible at the bottom of the left *cul-de-sac* and along the left wall of the vagina. This tumor, which is about the size of a small pea, can be felt through the abdominal wall. The posterior lip of the cervix is irregular and rugose. The anterior lip presents a hard tumor as large as a walnut. The os allows the insertion of the finger, and its edges are irregular. The uterus is movable and anteflexed. The body can be felt bimanually through the anterior vaginal wall. The inguinal glands are engorged. The patient is pale and emaciated. Through the speculum there is seen upon the anterior lip of the cervix an irregular tumor of a reddish yellow color, of the size of a large walnut, distinctly separate from the wall of the vagina. The posterior lip of normal size presents on one side an infiltrated portion of the same color and rugose aspect. The depth of the uterus is three and a half inches. The cervical canal is of normal size. The tumor bleeds slightly, and there is slight hemorrhage when the sound is passed. The uterus is movable and can be drawn down. The *culs-de-sac* are intact. The secretions are not fetid. On the 5th of May an operation was performed under chloroform, with the patient in the dorsal position. The posterior wall of the vagina was retracted by a Sims' speculum and the lateral walls held apart by retractors. The bladder was protected by a catheter and the womb drawn down by forceps. An incision was made through the vaginal wall at some distance from the border of the tumor and the lower part of the neck separated by the handle of a scalpel. The ablation of the tumor was performed with Paquelin's cautery and the infiltrated portion of the posterior lip was removed. The mucous membrane of the cervical canal was cauterized with the thermo-cautery. The mucous membrane of the two sides was united before and behind by two silk sutures. A small tampon saturated with perchloride of iron was applied to the cauterized surface, and the vagina packed with the iodoform gauze. Microscopical examination of the tumor showed its cancerous nature.

*May 10.*—Uterus anteflexed. In the roof of the vagina instead of the os uteri, was an excavation with irregular borders. The tumors on either side of the uterus had undergone no change. The infiltrated portion of the excavation was scraped with the sharp curette, and tampons were applied saturated with a solution of chloride of zinc one to ten.

*June 2.*—Uterus three inches deep, otherwise no change since May 20. On the 9th of June, the tumors on either side of the uterus seemed somewhat diminished in size; the cavity in the roof of the vagina had undergone considerable contraction and the vaginal mucous membrane looked healthy. The visible portion of the cervical mucous membrane was of a reddish yellow color, bleeding very little and hard to the touch. Depth of the uterus, three inches. At this time, as the patient seemed to be relieved, she left the hospital.

According to the statements of her husband, the condition of this patient has been very feeble since April, 1886. She often vomited and had severe pains, which were controlled only by injections of morphine. On the 13th of August, 1886, she was seized with violent convulsions and remained unconscious until her death, on the 20th of August, 1886.

We have here a spontaneous labor in a case of carcinoma of the neck of the womb. These cases are comparatively rare. It is evident that the degeneration of the neck of the womb leads in most cases to changes which present an obstacle to spontaneous labor, which the unaided powers of nature cannot overcome. The part invaded by the tumor loses its softness and distensibility, as well as its capacity for dilatation by the amniotic sac or by the presenting part of the fœtus, resulting in ruptures of the neck and of the lower portion of the uterus. Or else labor causes such injury to the tumor, followed by hemorrhage, that the artificial termination of labor becomes necessary. The pressure of the tumor may cause gangrene leading to death during the puerperium. If the tumor is confined to a portion of the cervix, or if its histological structure renders it less predisposed to laceration, the labor may be terminated spontaneously. The part which is intact compensates for the loss of distensibility in the part occupied by the tumor, or else the descending fœtus exerts upon the degenerated part sufficient pressure to prevent serious hemorrhage.

Some observers have maintained that the course of labor varies according as the tumor is located upon the anterior or the posterior lip. According to Cazeaux (*Traite Theorique et Pratique de l'art des accouchements, 8e edit., par Tarnier, Paris, 1870, page 722,*) labor takes place more easily in case of carcinomatous degeneration of the posterior lip. Cohnstein (*Ueber die Complication der Schwangerschaft und Geburts mit Gebarmutter-Krebs, Archiv. f. Gyn., Bd. V., page 366*) who has collected observations of cases of labor complicated by cancer of the neck of the uterus, has also found a greater number of spontaneous labors in cases of carcinoma of the posterior lip than of the anterior lip.

As long as the cancer is confined to a single lip, whether the anterior or the posterior, spontaneous labor is the rule and it sometimes is terminated easily and rapidly, as in the case which I have reported, dilatation usually taking place at the expense of the healthy portion of the cevix.

An important question presents itself in regard to cancer of the uterus. Is it possible and necessary to treat the cancer actively during pregnancy? Some gynecologists have recommended to remove the degenerated portion, while others prefer the expectant plan during pregnancy and an operation, if possible, later on.

In the presence of a disease which will certainly prove fatal to the mother at some time not very far off, the first thought should be of the safety of the child. Operations performed upon the genital organs during pregnancy often result in the interruption of pregnancy, and we have seen, on the other hand, that cancer confined to one lip very often gives results favorable to both mother and child.

It is my opinion that the treatment adopted in this case was perfectly justifiable, the expectant plan during pregnancy and an operation only after delivery. The operation delayed but did not prevent death.

